# The Gap Between Expected and Perceived Organizational Culture in an Iranian University of Medical Sciences from the Perspective of Different Stakeholder Groups

**DOI:** 10.31661/gmj.v13i.2507

**Published:** 2024-02-08

**Authors:** Mina Riahi, Seyed Ahmad Ahmadi, Aidin Aryankhesal

**Affiliations:** ^1^ Department of Health Services Management, School of Health Management and Information Sciences, Iran University of Medical Sciences, Tehran, Iran; ^2^ Health Management and Economics Research Centre, Iran University of Medical Sciences, Tehran, Iran

**Keywords:** Organizational Culture, University of Medical Sciences, Gap, Expectations, Perception, Stakeholder

## Abstract

Background: Organizational culture plays a key role in the success of organizations. Therefore, it is important to measure the gap between stakeholders’ perceptions and expectations of the dominant culture in that organization. This study investigates the gap between the perceptions and expectations of the academic community at the Iran University of Medical Sciences. Materials and Methods: This descriptive cross-sectional study was conducted from April to the end of October 2021. 960 students, faculty members, staff, and administrators were selected using simple random sampling. The data was collected through Robbin’s organizational culture questionnaire including ten components to assess the gap between the existing (perceived) and desired (expected) organizational status. Results: The highest and lowest gap between expectations and perceptions out of five possible scores were observed in the reward system (1.74±1.16) and communication patterns (1.01±1.16) components, respectively. The gap between participants’ perceptions and expectations is associated with individuals’ demographic characteristics in most organizational culture components (P0.05). Conclusion: Increasing productivity in the university depends on strengthening and promoting its organizational culture. The organizational culture can be improved by meeting the academic community’s expectations by paying attention to the demographic characteristics of staff and taking the necessary actions. Therefore, it should be a strategic priority for managers.

## Introduction

Since 2000, organizational culture has been at the center of the debate over the structure of healthcare reform among high-income countries including the United States and the United Kingdom. Policymakers and managers have recognized that structural reform alone cannot lead to desired changes in the health system [1]. Meanwhile, low and middle-income countries including Nigeria [2], Taiwan [3], Brazil [4], Ghana [5], Greece [6], and Iran [7][8][9][10], also understood the importance of organizational culture in the success or failure of reforms. The study that was conducted in British organizations shows that only 17% of employees are fully engaged, talented, and motivated, 63% perform normally and within the minimum standards and expectations, and the remaining 20% are dissatisfied and upset [1].

In developing countries, including Iran, the statistics of the third group are much higher [11]. Universities of medical sciences in Iran as part of the health system have educational, research, health, and medical duties which is based on their mission and function. Also, they have several healthcare centers and hospitals. The staff of these centers are employed by the universities of medical sciences. They are responsible for attracting, training, and educating specialized human resources, producing science and technology and providing health services to the cover community at all levels.

Due to the diversity of tasks, the organizational culture in these institutions becomes very complex. Each functional aspect of these institutions, including research, education, health care, and monitoring, requires its own organizational culture. Currently, these universities face challenges including inefficiency in establishing proper cooperation and organizing effective elements in the health sector, lack of training in skills and techniques in modern management, lack of fair distribution of resources, lack of strengthening educational and research policies to provide quality services and lack of selection of efficient managers [12][13].

On the other hand, the low or average score of organizational culture factors about knowledge management and failure to perform social and cultural functions indicate unfavorable conditions in universities. Hence, universities must adopt different strategies to achieve their goals. One of these strategies is to evaluate the gap between the current and desired state of organizational culture and provide solutions for it. Also, considering insufficient studies on the universities’ organizational culture and its importancein organizational productivity, the current study was conducted to evaluate the gap between staff expectations and perceptions at the Iran University of Medical Sciences.

## Materials and Methods

**Table:** Table1. Demographic Characteristics of the Subjects

**Variable**	**Grouping**	**Frequency/ (percentage)**	**Variable**	**Grouping**	**Frequency/ (percentage)**
**Gender**	Male	371(38.6)	*Employment Status	Formal	341(35.5)
	Female	589(61.4)		Temporary	35(3.6)
**Marital status**	Married	571(59.5)		Under a contract	54(5.6)
	Single	389(40.5)		Contractual	213(22.2)
**Age**	Less than 30 years	181(18.9)		The rest	52(5.4)
	31-40	370(38.5)	*Work Experience	Less than 5 years	92(9.6)
	41-50	285(29.7)		5-10	93(9.7)
	More than 50 years	124(12.9)		10-15	154(16)
**Level of education**	Diploma and less than diploma	58(6)		15-20	150(15.6)
	Associate Degree	42(4.4)		20-25	75(7.8)
	Bachelor’s Degree	220(22.9)		25-30	131(13.6)
	Master of Science	370(38.5)	**Management experience	Less than 5 years of	18(1.9)
	Ph.D.	270(28.1)		5-10	44(4.6)
**Field of study**	Medicine	237(24.7)		10-15	29(3)
	Health	116(12.1)		15-20	12(1.3)
	Paramedical	69(7.2)		20-25	9(0.9)
	***Science	162(16.9)		25-30	3(0.3)
Humanities	117(12.2)	**Education** **/** **Service place**		Hospitals	42(4.4)
	Engineering	60(6.3)		Colleges	508 (52.9)
	Other medical sciences	199(20.7)		Deputies	318(33.1)
				Health care centers	82(8.5)
				University department	10 (1)

^*^ The statistical population includes faculty members, managers and employees. ^**^ The statistical community includes managers. ^***^ This study includes: Mathematics and Statistics, Chemistry, Environment, Management and Economics, Biology and Language.

This descriptive cross-sectional study was conducted from April to the end of October 2021 at the Iran University of Medical Sciences located in Tehran, Iran.

Population and Sample Size

The university community included faculty members, managers, employees, and students. The stratified sampling method was used to increase the similarity between the sample and the community. The sample size was determined based on Morgan’s Chart.

Data Collection Tool

The data collection tool was Robbin’s international model questionnaire. The questionnaire consisted of ten components including a reward system, control, risk-taking, leadership, management support, conflict, communication, identity, integration, and initiative [14]. The Persian version of the Robbins questionnaire in the study of Raste Moghadam et al. [15] was used because of is compatible with Iran’s culture. The face validity and reliability of the questionnaire in the current and favorable conditions of organizational culture were confirmed with Cronbach’s alpha of 92%. The questionnaire consisted of 68 questions and was evaluated for both existing and desirable situations of organizational culture. The organizational culture refers to the ten components, each one is based on a 5-point Likert scale on a spectrum with a very low to a very high range and the average score of each component indicates the status of organizational culture.

Data Collection Method

Data were collected via distributing questionnaires manually in the first stage and then by sending electronic questionnaires via email and social network applications (WhatsApp and Telegram) due to Coronavirus disease 2019 (COVID-19).

Statistical Analysis

Descriptive statistics were used to analyze the relation between the demographic characteristics of individuals and components of the organizational culture. Considering the normality of data distribution, the Analysis of Variance (ANOVA) test was used to determine the mean and standard deviation of the components in the current and desired situation of the organizational culture, the difference between the current and desired status, and their relation to employment status and service/education place. Independent-Samples T-Test was used to determine the relation of components with gender and marital status. Pearson correlation test was applied to determine the relation between components and age. We used the Spearman correlation test to determine the relation of components with education, management, and work experience. In all tests, a standard level of 5% and a confidence interval of 95% were considered. SPSS software version 25 made by International Business Machines Corporation (IBM) company in the United States (available on the Web at www.ibm.com/legal/copytrade.shtml) was used for data analysis.

Ethical Considerations

In all stages of the study, the critical condition of the COVID-19 pandemic, including compliance with health protocols, social distancing, using the electronic questionnaires due to the absence of individuals, and confidentiality of the participants’ information was considered. The protocol of this study was approved in 2021 with the ethics code IR. IUMS.REC1400.114 of the National Center for Ethics in Iranian Biomedical Research.

## Results

**Table:** Table2. Significance Level and Correlation Coefficient of Organizational Culture Components in Relation to Variables

Component		Reward system	Initiative	Risk taking	leadership	Integrity	Management support	Control	Identity	Communication	Conflict
Variable											
Employment Status		0.097	0.157	0.012	0.178	0.002	0.01	0.459	0.214	0.855	
Service/Education place			0.32	0.012	0.001	0.111		0.747		0.001	0.434
Gender		0.335	0.197	0.969	0.478	0.859	0.335	0.724	0.779	0.843	0.317
Marital status		0.119	0.402	0.781	0.551	0.652	0.615	0.819	0.696	0.167	0.171
Age	R	**0.207	**0.11	**0.168	**0.14	**0.112	**0.153	*0.075	**0.126	*0.082	0.025
	P-value		0.001			0.001	0.021		0.011	0.444
Level of education	R	**-0.093	0.009	0.022	0.014	0.047	-0.061	0.003	-0.023	-0.063*	**0.125
	P-value	0.004	0.775	0.487	0.664	0.145	0.057	0.931	0.476	0.05	
Work experience	R	0.101	*0.036	0.072	0.064	*0.046	0.071	*0.036	0.083	*0.047	*-0.026
	P-value	0.007	0.339	0.055	0.091	0.222	0.061	0.346	0.028	0.213	0.487
Management experience	R	-0.128	-0.11	-0.184	*0.022	-0.083	-0.03	0.012	*-0.024	*0.043	-0.063
P-value	0.169	0.24	0.048	0.812	0.376	0.753	0.859	0.8	0.649	0.503


^*^ Correlation is significant at the 0.01 level (2-tailed) ^**^ Correlation is significant at the 0.05 level (2-tailed)

Due to some participants’ unwillingness to answer the questions, attitudinal complexities in social studies, and the necessity of social distancing due to COVID-19pandemic, the response rate of the questionnaires was 85%. Out of 1,127 distributed questionnaires, 960 were returned. Of the participants, 102 (10.6%) were faculty members, 265 (27.6%) students, 115 (12%) managers, and 478 (49.8%) employees. The demographic characteristics of the subjects are shown in Table-1.

**Figure-1:**
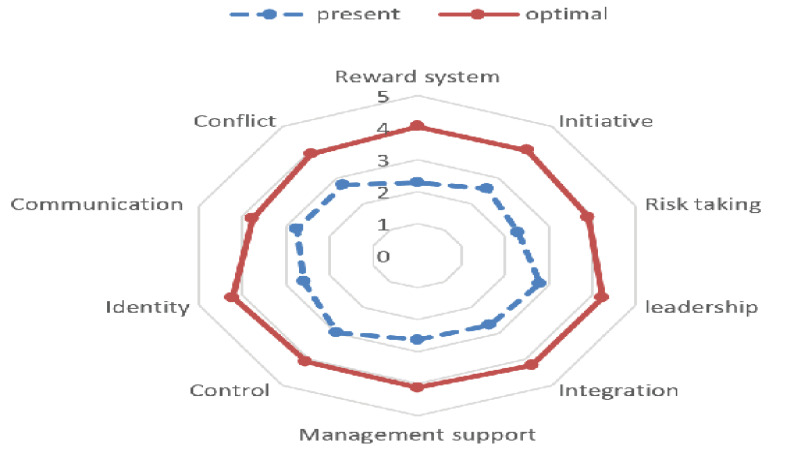
Figure 1. Comparison of the current and desired status of organizational culture from the perspective of the subjects

From the perspective of the participants in this study, the elements of organizational culture in the current situation are below expectations. Based on the average gap between the desired situation and the current situation; the highest to lowest gaps (mean and standard deviation out of 5 possible scores) were related to the components of the reward system (1.74±1.16), identity (1.61±0.97), risk-taking (1.60±1.07), integration (1.56±1.09), management support (1.49±1.08), initiative (1.47±1.10), leadership (1.42±1.01), conflict (1.19±1.13), control (1.13±0.93), and communication (1.01±1.16) which were expressed significantly (Figure-1). The level of significance and correlation coefficients of organizational culture components with demographic variables are shown in Table-2. Based on the ANOVA test there was sa ignificant relation between the employment status variable and the mean gap in the components of risk-taking, integrity, management support, and conflict, and between the service/education place variable with the mean gap in the components of the reward system, risk-taking, leadership, management support, communication, and identity (P<0.05, Table-2). The highest mean gap in these components was observed in official employment status and health centers, directorates, colleges, and hospitals, respectively. According to Independent-Samples T-Test, there was no significant relation between organizational culture, gender, and marital status (P>0.05). According to Pearson’s analysis, there was a positive correlation between the age variable and all components, which was significantly expressed in all components except conflict. In Spearman correlation analysis, there was an inverse correlation between the education level of communication and reward system, and there was a positive correlation in the conflict component which was expressed as significant. A significant positive correlation between work experience and the reward system and identity was observed. Between management experience and risk taking, there was clear as a significant inverse correlation (Table-2).

## Discussion

Managers of health organizations, like others must have an implicit understanding and awareness of the beliefs and values that prevail in the organization which shape the behavior of employees and motivate them [16]. If we consider the health indicators, we realize that the low level of many of these provides a weak culture in these organizations. The results of this study showed the weakness of organizational culture at different levels of this organization in the form of 10 components. According to the existing gap, it seems that the payment of rewards is not based on people’s performance and other criteria are considered. The results in studies of Ahmadi et al. [17], Hedayati et al. [18], Blouin et al. [19], and Rovithis et al. [6] showed weakness in the reward system of the studied organizations. Considering that the reward system is part of the motivational system in organizations motivating employees to achieve organizational goals, it is necessary to consider a mechanism to correct the process of the reward system.

Based on the results, the community does not consider their membership as a cause of pride and does not feel proud of working in this organization. Employees feel that their professional and organizational role and identity in the challenge of technology is limited compared to the other professions. Ahmadi et al. stated that the comparison of the average score of the answers with the hypothetical average of three shows that the level of identity in the studied sample is lower than the average level [17]. According to Kerfoot, strengthening the identity and creating a common vision among group members lead to better leadership performance [20].

If the identity is not strengthened in people; the commitment, loyalty, responsibility, durability, and hard work of the employees will be lost. According to the gap in the risk-taking component, it can be said that people emphasize the internal stability of the present situation and make no effort to change it. Zazzali et al. [21] and Acar et al. [22] reported in their studied organizations that people do not believe in risk acceptance to create a creative and dynamic environment. In dynamic organizations where creativity, competition, growth, and development are important, the risk tolerance is high.

A low spirit of individual independence as one of the organizational culture elements will lead to organizational efficiency reduction [16]. The gap in the component of integration indicates the low spirit of cooperation and collaboration in this organization. Ahmadi et al. [17], Hedayati et al. [18], Beatrice et al. [23] stated that the unity and integrity of groups to join each other to achieve common goals is not possible in a desirable way. The mission, goals, and strategies of the organization should not be in tension with the organizational culture [24] and to strengthen organizational effectiveness, the members of the organization need to work together.

The index of management support in this organization is far from the desired level, which was in line with the studies of Mossadegh Rad et al. [25], and Zachariadou et al. [26]. But, Vazife et al. [27] and Yaman et al. [28] stated that the dominant culture in the organization was nurturing and protective. Given that the highest correlation in organizational culture was observed between employee satisfaction and management support for employees [17][23][26][29]. Managers should communicate with their subordinates, help them, and support them. It can be said that in this organization there was no freedom of action or appropriate space for expressing thoughts and presenting new proposals by individuals. These results were also reported in the studies of Dargahi et al. [16], Zazzali et al. [21], and Abass et al. [30]. Organizational culture as a facilitator and motivator, directly and indirectly, affects the creativity of employees to achieve success through empowerment, innovation, and creativity which are the highest motivational factors in each individual [31]. The university culture must move towards a clan and adhocracy organizational culture in which innovation and creativity are strengthened based on market demand.

While the leaders play a key role in promoting and maintaining organizational culture and creating an appropriate cultural structure within the organizations, the results show that the leadership component is far from the desired situation. Dejong believes that leadership is one of the indicators of organizational culture and plays an important role in the excellence of organizational performance of hospitals [32].

Transformational leadership behaviors create a culture in the organization in which initiative, creativity, and empowerment of employees are under their support so that the leaders of these organizations are more successful in acquiring knowledge and analyzing information and complex situations [33]. In this organization, people refrained from expressing opinions and suggestions that were contrary to those of their managers. Conflict with colleagues, frequent contact with patient’s suffering and death, employing a person in a job contrary to his abilities and information, and lack of support resources are the causes of stress in employees; especially in hospitals [34] and health centers. These results were also reported in the studies of Dargahi et al. [16], Mash et al. [35], and Beatrice et al. [23]. The mismatch of members’ norms and individual values with organizational values weakens the organizational culture and creates problems including job dissatisfaction and reduced motivation and performance of individuals. There is direct control and supervision within the framework of laws and requirements in this organization. Ashena et al. [36] and Acar et al. [22] also stated that in their studied organizations individual decisions were limited and employees acted according to established norms. Bureaucratic culture does not create the necessary flexibility in the organization and leads to habituation to existing procedures and unwillingness to innovate and create new ideas in staff [27].

On the other hand, the staff refuse to share their knowledge with others and turn that knowledge into skills that can be effective in solving the problems of the organization. According to the results of the exchange of information, reports, concepts and feelings that are relatively appropriate between employees and formal communication patterns refers to explicitness. Ahmadi et al. [17] and Rider et al. [29] also reported that the amount of communication in the studied sample was lower than average. However, Armstrong’s et al. [37] study was not in line with this study because the market culture dominates the organization. The Joint Commission for the Accreditation of Health Care Providers (JCAHO) believes that the lack of proper communication patterns is responsible for 85% of the incidents and errors occurring while employees are on duty [38]. Therefore, to promote organizational culture in the university, it is a priority for managers to strengthen the components of organizational culture.

The results showed that different employment statuses in the organization have created different motivations for people. Individuals with formal employment status showed higher expectations due to their awareness and longevity in the organization which should be the attention of managers. To improve the organizational culture, attention also needs to be paid to subcultures at all organizational levels.

The results of the study showed that the gender and marital status of people do not influence the organizational culture. Mehdizadeh et al. [39] and Ebrazah et al. [40] also stated that organizational culture is the same between the two genders. However, Rashedi et al. [41] stated that people showed different organizational cultures according to their gender characteristics.

These results show the difference in people’s views in every other organization. With increasing age, the mean gap has increased in all components except conflict. With increasing work experience, the mean gap has increased in the reward system and identity. It can be said that with increasing age and work experience, everyone considers the organization as their own and uses their experiences to advance the organization’s goals.

Therefore, they expect to be seen and compensated for their service. The failure of managers to pay attention to these expectations causes dissatisfaction, low performance, and the desire to leave the organization in the university community.

With the increase in management experience, the tendency to take risks in managers has decreased. Therefore, it can be said that with the increase in their management experience, managers demand more stability and a sense of security in the environment and are less likely to seek risks and stress. With the increase in the level of education, the average gap in the components of the conflict has increased but it has decreased in communication and the reward system. As their educational level grows, the expression of their criticisms and disagreements will be revealed openly. Also, due to their scientific capabilities, they have more relation and the prevailing organizational conditions; so, they do not have more expectations regarding their reward system. Studies related to these findings were not found. Thus, the authorities should consider the factors influencing the components to improve the situation of organizational culture in the university.

## Conclusion

The existing gap between people’s perceptions and their expectations of organizational culture can disrupt the organization’s management system. It is possible to reduce or eliminate this gap by considering the role of demographic variables of people on the components of organizational culture, as well as the correlation between the components. Considering the comprehensiveness of this study for accomplishing these surveys in distinction from the other studies, it is suggested that the results of this study can be used as a tool for managers and policymakers in creating environments with a favorable organizational culture by creating cultural changes, changing management styles and empowering the members of the organization. Also, it is suggested to use the business of successful organizations in the world to promote and strengthen the organizational culture of the university, taking into account the cultural, social, and economic conditions of the country.

## Acknowledgment

Iran University of Medical Sciences supported this research financially (Grant number: IUMS/SHMIS_013720023).

## Conflict of Interest

The authors declare no conflict of interest.
